# Quiet quitting among Gen Z and millennial employees: contributing factors and organisational mitigation strategies

**DOI:** 10.3389/fpsyg.2026.1839119

**Published:** 2026-07-24

**Authors:** Marilyn Joe, Nelesh Dhanpat

**Affiliations:** 1 University of Johannesburg College of Business and Economics, Johannesburg, South Africa; 2 University of Johannesburg, Johannesburg, South Africa

**Keywords:** employee disengagement, Gen Z, millennials, quiet quitting, retention strategies, South Africa, workplace culture

## Abstract

**Introduction:**

Quiet quitting, whereby employees limit their effort to the minimum requirements of their job descriptions, represents an emerging form of voluntary disengagement that is particularly prevalent amongst Gen Z and Millennial employees. Despite growing global attention to this phenomenon, limited research addresses its contributing factors within the South African workplace context.

**Method:**

This study employed an interpretivist qualitative approach using purposive and snowball sampling to conduct semi-structured interviews with 12 South African Gen Z and Millennial employees across various professional sectors. Data were analysed using Braun and Clarke’s reflexive thematic analysis framework.

**Results:**

Three key themes emerged: (1) quiet-quitting behaviours and attitudes, encompassing withdrawal, minimal effort performance, career transition preparation, generational work attitudes, and digital distraction; (2) workplace value misalignment, encompassing inadequate compensation and recognition, limited growth opportunities, changing generational priorities, and toxic work environments; and (3) organisational strategies to mitigate quiet quitting, encompassing workplace culture and voice, detection and intervention, growth and development opportunities, well-being and work-life balance, and recognition and reward systems.

**Discussion:**

The study contributes to theory by proposing a generational refinement of job embeddedness theory and introducing a motivational typology distinguishing protective and assertive orientations to quiet quitting. The findings suggest that, within this sample, quiet quitting amongst younger employees appears to be driven primarily by a perceived misalignment between employee values and organisational culture and practices, though the transferability of these patterns to other contexts requires further investigation. To address this, organisations could consider implementing strategies that foster inclusive cultures, provide development opportunities, and improve recognition systems. HR professionals and leaders may use these findings to implement targeted interventions to reduce disengagement and turnover risk amongst Gen Z and Millennial employees in South African workplaces.

## 1 Introduction

Employee turnover represents a critical challenge for contemporary organisations (Lim and Parker, 2020), with voluntary departures carrying particularly high costs in terms of morale, productivity, and financial performance (Al-Suraihi et al., 2021; Dhanpat et al., 2018; Zhen and Mansor, 2020). Employee turnover can be categorised into voluntary and involuntary types (Rijamampianina, 2015), with voluntary turnover often posing greater challenges for organisations because of its impact on employee morale, productivity, and financial outcomes (Hamouche et al., 2023; Johar et al., 2023; Lestari et al., 2024). Voluntary turnover occurs when employees leave organisations of their own accord (Rijamampianina, 2015), whereas involuntary turnover arises when management asks employees to exit the organisation (Belete, 2018). Research in the South African context confirms that voluntary turnover carries significant operational and human costs across sectors including retail, healthcare, and mining (Ngqeza and Dhanpat, 2021; Dhanpat et al., 2018).

Traditional research on voluntary turnover has primarily focused on overt forms of resignation, in which employees provide formal notice before departing from their roles (Mokoena et al., 2022; Schlechter et al., 2016). There is, however, increasing recognition of a more subtle form of voluntary turnover, known as quiet quitting, which is particularly prevalent amongst Generation Z (Gen Z) and Millennial employees (Shah and Parekh, 2023; Veren et al., 2025). Quiet quitting refers to situations in which employees strictly adhere to the minimum expectations outlined in their job descriptions (Mahand and Caldwell, 2023; Serenko, 2024).

Quiet quitting often stems from the need for work-life balance, low trust, inadequate pay, poor communication, and excessive workloads (Selesho and Matjie, 2024; Yikilmaz, 2023). In the current context of high unemployment rates, employees may opt for quiet quitting to retain job security (Bush, 2022). Recent surveys suggest that quiet quitting affects approximately 50% of the global workforce (Smith, 2022), with particularly high prevalence amongst younger employees.

Unlike traditional turnover, quiet quitting may not be immediately apparent to managers or colleagues, which makes it difficult for organisations to address and mitigate its underlying causes. Whilst traditional quitting involves employees actively leaving their roles (Detert, 2023; Galanis et al., 2023), quiet quitting represents a passive approach in which employees remain in their roles but express dissatisfaction by limiting their efforts to the bare minimum required (Galanis et al., 2023). Traditional quitting is therefore reactive, as employees take action to leave, whilst quiet quitting is passive, as employees refrain from acting beyond their minimum obligations.

In South Africa, where youth unemployment exceeds 60% (Statistics South Africa, 2025), understanding retention dynamics amongst younger workers has become critical. To date, there is limited research on quiet quitting in the South African workplace context, particularly in relation to the Gen Z and Millennial workforce (Bangura and Lourens, 2023; Chitamba, 2025; Manyaga, 2024; Utete et al., 2023). Most prior studies have focused on voluntary turnover amongst earlier generations (Hollman and Luthans, 2020; Ivanović and Ivančević, 2019).

This study addresses this gap by examining: (1) factors contributing to quiet quitting amongst South African Gen Z and Millennial employees, (2) how these factors manifest in workplace behaviours, and (3) organisational strategies to mitigate disengagement. By focusing on the South African context, where limited research exists, the study contributes practical insights for managing multigenerational workforces in emerging economies.

### 1.1 Conceptual clarification: quiet quitting and related constructs

It is important to distinguish quiet quitting from related but conceptually distinct workplace phenomena. Employee disengagement, as conceptualised by Kahn (1990), refers to the withdrawal of cognitive, emotional, and physical energies from work role performances. Whilst quiet quitting shares features with disengagement, it is distinguished by its intentional and strategic nature: quiet-quitting employees consciously calibrate their effort to contractual requirements rather than experiencing a diffuse loss of motivation. Burnout, defined as a state of chronic exhaustion, cynicism, and reduced professional efficacy (Maslach and Leiter, 2016), represents a health-related outcome of prolonged occupational stress. Although burnout may precipitate quiet quitting, the two constructs differ in that burnout is an involuntary psychological state whereas quiet quitting involves a deliberate behavioural choice. Quiet quitting also differs from organisational withdrawal behaviours such as absenteeism and lateness (Skorupko and Zieba, 2026), in that quiet-quitting employees maintain physical presence and fulfil minimum role requirements. Finally, quiet quitting is conceptually adjacent to, but distinct from, organisational citizenship behaviour (OCB) withdrawal. Whereas OCB withdrawal involves the cessation of discretionary, extra-role behaviours (Organ, 1988), quiet quitting encompasses a broader attitudinal reorientation that may include psychological detachment, identity decoupling from the work role, and active boundary-setting. In this study, quiet quitting is defined as the deliberate restriction of work effort to the minimum requirements specified in one’s job description, accompanied by psychological disengagement from discretionary contribution, driven by perceived misalignment between employee expectations and organisational conditions.

## 2 Theoretical background and literature review

Understanding quiet quitting requires examination of both individual-level factors influencing workplace attachment and the generational characteristics that shape employee expectations. The practice of quiet quitting is particularly prevalent amongst young employees aged 18 to 35 years (Shah and Parekh, 2023; Taufik et al., 2024). This age range encompasses two generational cohorts: Gen Z (born between 1997 and 2012) and Millennials (born between 1981 and 1996) (Vacalares et al., 2023). These individuals are likely to encounter difficulties achieving a work-life balance owing to gig work, digital connectivity, and evolving career expectations (Shah and Parekh, 2023; Veluchamy et al., 2021).

Notwithstanding the extensive research on the factors contributing to employee retention (Dhanpat and de Braine, 2022; Chatzoudes and Chatzoglou, 2022; Mahadi et al., 2020) and commitment (Megawaty et al., 2022; Saputra and Mahaputra, 2022; Vuong et al., 2020), there is limited qualitative research on the recent phenomenon of quiet quitting amongst Gen Z and Millennial employees. It is important to explore quiet quitting in the workplace because it is difficult to detect until its repercussions, such as increased employee turnover rates, poor customer service, and inferior work quality, become apparent (Jelača and Golubović, 2024; Johar et al., 2023; Kachhap and Singh, 2024). There is therefore a need for early-detection strategies tailored to Gen Z and Millennial cohorts.

### 2.1 Theoretical perspectives on quiet quitting

#### 2.1.1 Job embeddedness theory

Job embeddedness theory (Mitchell et al., 2001) suggests that employees remain with organisations owing to three interconnected factors: fit (compatibility between employee and organisation), links (formal and informal connections), and sacrifice (perceived costs of leaving). Unlike traditional turnover models that focus on job satisfaction, job embeddedness explains why employees stay despite dissatisfaction (Lee et al., 2008).

This framework illuminates quiet quitting by revealing how employees may remain physically present whilst psychologically withdrawing (Atiq et al., 2025). Recent empirical evidence supports this, demonstrating that quiet quitting manifests as a distinct form of workplace withdrawal in which employees are physically present but mentally and emotionally disengaged, driven by psychological processes including work alienation, perceptions of unfairness, and unmet expectations (Georgiadou et al., 2025; Almarzoqee et al., 2025). Understanding unfairness and unmet expectations helps identify which factors contribute to disengagement amongst younger workers. This resonates with earlier South African evidence suggesting that the psychological contract, specifically perceptions of fairness and reciprocity, plays a foundational role in shaping employee retention decisions (Dhanpat et al., 2018).

#### 2.1.2 Theory of generations

The theory of generations, originating with Mannheim (1952), indicates that cohorts experiencing similar historical events during formative years develop shared values and behaviours. Contemporary research has identified distinct workplace preferences amongst generations (Lyons and Kuron, 2014).

Millennials (1981 to 1996) entered the workforce during a period of technological expansion and economic instability, developing preferences for flexibility, purpose-driven work, and frequent feedback (Myers and Sadaghiani, 2010). Gen Z (1997 to 2012), as digital natives raised during economic uncertainty and social activism, prioritise work-life boundaries, authenticity, and social responsibility even more strongly (Seemiller and Grace, 2016; Schroth, 2019).

These generational characteristics suggest that quiet quitting may represent a generational response to traditional workplace structures. When organisations fail to align with younger employees’ values, particularly regarding boundaries, purpose, and authenticity, these workers may disengage rather than conform (Twenge, 2010). This theoretical lens helps explain why quiet quitting appears particularly prevalent amongst Gen Z and Millennials.

### 2.2 Factors contributing to quiet quitting

#### 2.2.1 Compensation, recognition, and career development

The relationship between compensation, recognition, and quiet quitting reflects a broader pattern of perceived reciprocity failure. Employees who experience a sustained imbalance between their contributions and the rewards they receive tend to recalibrate their effort downward, a pattern consistent with social exchange theory (Blau, 2017) and empirical evidence from multiple contexts (Detert, 2023; Serenko, 2024; Sitopu et al., 2020). This recalibration is not uniform across generations, however. Whilst financial compensation remains salient for younger workers, particularly in high-cost urban environments, recent evidence suggests that Gen Z employees weigh intrinsic rewards, including recognition, purpose, and developmental feedback, at least as heavily as extrinsic compensation (Krishna and Agrawal, 2024; Mabaso and Mathebula, 2025). Recognition effectiveness itself appears to vary generationally: Millennials tend to value public acknowledgement, whilst Gen Z employees may prefer private, authentic feedback (Krishna and Agrawal, 2024). These differential preferences imply that a one-size-fits-all compensation and recognition strategy may inadvertently contribute to the very disengagement it seeks to prevent.

Research consistently demonstrates that perceived career stagnation, characterised by limited advancement opportunities and skills development, drives employee disengagement (Pagayanan, 2021; Shabbir et al., 2020; Xueyun et al., 2023). Evidence from the South African context suggests that meaningful work and opportunities for growth are central to early-career professional engagement (Dhanpat et al., 2025), and that proactive work behaviours such as job crafting represent a constructive alternative to disengagement when employees seek greater purpose (Dhanpat and de Braine, 2022).

Perspectives differ on accountability: whilst some scholars emphasise organisational responsibility for providing growth pathways (Xueyun et al., 2023), others argue that employees should demonstrate proactivity in self-development (Ellera et al., 2023). For younger workers entering flattened organisational structures, limited advancement prospects may be particularly demotivating, potentially accelerating quiet-quitting behaviours.

Quiet quitting stems from an imbalance between inputs and outputs, whereby employees feel that they are giving more than they are receiving in return for their work (Detert, 2023). When employees feel as though they are not benefiting from their efforts, they develop an ‘equal pay for equal work’ attitude. Employees are motivated when they are rewarded generously for their contributions (Sitopu et al., 2020). When employees perceive their pay to be stagnant, they tend to be demotivated and unwilling to go the extra mile (Rosłon, 2020). By quiet quitting, employees do the bare minimum with the aim of preserving their wage, sometimes described as ‘acting their wage’, because they are concerned about losing their job (Hamouche et al., 2023).

#### 2.2.2 Work environment, leadership, and inclusion

Toxic workplace cultures, characterised by bullying, harassment, and destructive leadership practices, represent powerful drivers of quiet quitting (George, 2023; Thakur, 2024). Such environments create psychological distress, prompting employees to establish protective boundaries by limiting workplace engagement (Armstrong and Pfandler, 2024; Duda and Drożdżyńska, 2024). For Gen Z employees, who report lower tolerance for toxic behaviour than previous generations (Seemiller and Grace, 2016), cultural toxicity may trigger particularly rapid disengagement. South African research on workplace bullying (Badenhorst and Botha, 2022) suggests these dynamics warrant particular attention in contexts with hierarchical organisational cultures. Research indicates that manager credibility and strategic alignment are significant determinants of employee motivation (Lees and Dhanpat, 2021), and that leadership quality directly shapes performance outcomes (Mokgahla et al., 2015).

Management practices significantly influence employee engagement levels (Mdhlalose, 2025; Evans-Uzosike and Okatta, 2025). Micromanagement, characterised by excessive control and monitoring, undermines autonomy and trust, contributing to dissatisfaction, turnover, and quiet quitting (Kachhap and Singh, 2024; Ryan and Cross, 2024; Samakao and Mulenga, 2023). Conversely, transformational leadership styles that emphasise inspiration, support, and empowerment enhance engagement amongst younger workers (Bass and Riggio, 2006). The contrast between controlling and empowering management approaches appears particularly important for Gen Z and Millennial employees, who value autonomy and collaborative leadership (Barhate and Dirani, 2022).

Diversity and inclusion in the workplace refer to appreciating each employee’s distinct traits and viewpoints, and creating an environment in which all employees, regardless of their background, feel valued and encouraged (Bhatt et al., 2024; Kornau et al., 2023; van Bommel et al., 2024). Quiet quitting may be associated with a perceived lack of belonging, which could impede efforts to promote inclusion, diversity, and equity (Mahand and Caldwell, 2023; Pevec, 2023). Employees quiet-quit when they feel marginalised and unrecognised (Corbin and Flenady, 2024). Research indicates that perceptions of fairness and inclusion are closely linked to organisational citizenship behaviours, with employees who feel excluded less likely to contribute beyond their minimum obligations (Ngqeza and Dhanpat, 2022).

Unfavourable working conditions are frequently associated with quiet quitting (Mahand and Caldwell, 2023; Xueyun et al., 2023). Research suggests that high job demands, understaffing, and resource shortages contribute to quiet quitting (Galanis et al., 2024; Galanis et al., 2025). Employees who experience unfavourable working conditions tend to develop negative attitudes towards their work (Pathan, 2022; Ehrnrooth et al., 2021). Conversely, an employee is more likely to be satisfied when working in an environment that challenges them positively and provides pleasant conditions, which assists organisations in retaining talent (Dhanpat et al., 2018; Islam et al., 2024).

#### 2.2.3 Work-life balance and generational priorities

During the COVID-19 pandemic, employees were faced with heavy workloads for which they were not adequately compensated (Dhanpat and de Braine, 2022; Kang et al., 2023). South African research documents significant deterioration in employee well-being, heightened psychological distress, and disrupted work boundaries during and after the pandemic (Dhanpat and de Braine, 2022; Dhanpat et al., 2023). Post-pandemic mental health challenges in South African workplaces continue to affect employee engagement and retention (Dhanpat et al., 2025). Owing to work-life imbalances and difficulties managing personal challenges, some employees engage in quiet quitting (Efendi et al., 2023; Sarwar et al., 2024), which may involve establishing healthier work boundaries (Armstrong and Pfandler, 2024; Pratiwi et al., 2023). Quiet quitting is therefore sometimes exercised as a means of achieving work-life balance (Efendi et al., 2023; Ellera et al., 2023).

### 2.3 Conceptual framework

This study draws on two complementary theoretical perspectives to examine quiet quitting amongst Gen Z and Millennial employees. Job embeddedness theory (Mitchell et al., 2001) provides the structural lens through which employee attachment and disengagement are understood. Its three dimensions, namely fit, links, and sacrifice, offer a framework for analysing why employees remain in organisations despite dissatisfaction and, conversely, why low perceived fit or minimal sacrifice may facilitate psychological withdrawal rather than formal resignation. The theory of generations (Mannheim, 1952) complements this structural perspective by providing a contextual lens that accounts for the distinctive values, expectations, and workplace orientations of different generational cohorts. Together, these frameworks suggest that quiet quitting occurs at the intersection of structural misalignment (low fit, weak links, minimal sacrifice) and generationally shaped expectations regarding purpose, autonomy, recognition, and work-life boundaries. This integrated framework guided both the design of the interview schedule, which probed participants’ perceptions of organisational fit, workplace connections, and perceived costs of disengagement alongside generational values, and the analytical process, in which themes were interpreted through the dual lens of structural embeddedness and generational orientation.

### 2.4 Mitigating and retention strategies for quiet quitting

#### 2.4.1 Career development and mentorship

Quiet quitting may arise when employees believe that they have limited opportunities for career advancement (Xueyun et al., 2023). As younger generations transition into the workplace, they must acquire new skills and behaviours to navigate it successfully. Mentorship plays a critical role in the transfer of skills and knowledge in the workplace (Spence et al., 2018). Mentorship programmes demonstrably improve retention amongst early-career employees (Mahendran et al., 2022). In the South African context, graduate employees who employ self-management strategies demonstrate significantly higher work engagement (Dhanpat et al., 2021), and structured onboarding and talent development processes are foundational to embedding younger employees effectively (Dhanpat, 2021). Recent evidence further suggests that formal coaching programmes play a meaningful role in supporting early-career professionals through workplace transitions (Hoosen and Dhanpat, 2025). From a quiet-quitting perspective, mentorship can help younger employees integrate more effectively into organisations and may reduce the likelihood of disengagement. Younger workers particularly value developmental relationships (Wikstrom, 2023).

#### 2.4.2 Equal pay structures and employee recognition

To address quiet quitting, which may originate from perceptions of unequal pay distribution, organisations should implement transparent and equitable pay structures. Organisations may introduce reward and incentive schemes and competitive salaries to maintain employee satisfaction (Suhendar et al., 2023). Organisations may also implement piece-rate systems to reward employees for additional work by paying them according to their output (Harper et al., 2018), as well as bonus schemes to acknowledge and encourage high performance (Barinua et al., 2022).

Employee recognition involves acknowledging employees for their contributions and achievements (Kgarimetsa and Naidoo, 2024). Effective employee recognition leads to improved performance, lower turnover, and greater job satisfaction, thereby enhancing organisational success and profitability (Ampofo and Karatepe, 2022; Botha, 2023; Yang and Jiang, 2023). Recognition effectiveness depends on personalisation: whilst Millennials tend to value public acknowledgement, Gen Z employees may prefer private, authentic feedback (Krishna and Agrawal, 2024).

#### 2.4.3 Promoting work-life integration and work-life balance

Whilst work-life balance aims to establish an equilibrium between work and personal life in response to conflicting demands, work-life integration seeks to blend the elements of work and life more effectively (Singh and Mazhar, 2019). Failure to address work-life balance concerns can result in adverse consequences for organisations, including increased attrition, absenteeism, and pay-related disputes (Brough et al., 2008). Addressing work-life balance is therefore important in tackling quiet quitting, given its association with excessive workloads (Kang et al., 2023).

#### 2.4.4 Supportive work environment and leadership training

It is important to understand what drives Gen Z and Millennial employees and how the workplace can be adapted to accommodate their needs (Barhate and Dirani, 2022; Mahmoud et al., 2021). Studies suggest that diversity and inclusivity initiatives can help to alleviate quiet quitting (Mahand and Caldwell, 2023). This can be achieved by reassessing reporting structures and developing platforms for open communication to better retain the newer workforce (Campton et al., 2023). Developing a positive working environment as a means of reducing quiet quitting involves encouraging a collaborative and supportive culture and providing opportunities for interpersonal interaction so that employee needs and expectations can be understood (Hong et al., 2023; Kachhap and Singh, 2024).

Whilst existing research identifies multiple quiet-quitting determinants (Yikilmaz, 2023; Öztürk et al., 2023), several gaps remain. First, most studies originate from Western contexts (Bazargan et al., 2025; Mahand and Caldwell, 2023; Toska et al., 2025), which limits their applicability to emerging economies such as South Africa. Second, empirical evidence focuses predominantly on quantitative surveys rather than in-depth qualitative exploration of lived experiences. Third, few studies examine how contributing factors interact or vary between Gen Z and Millennial cohorts. Fourth, quiet quitting has not been sufficiently distinguished from adjacent constructs such as disengagement, burnout, and withdrawal (see Section 1.1), resulting in conceptual ambiguity that limits theoretical precision. This study addresses these gaps through qualitative investigation of South African Gen Z and Millennial employees’ quiet-quitting experiences, guided by an integrated conceptual framework (see Section 2.3) that draws on job embeddedness theory and the theory of generations to explore both contributing factors and effective mitigation strategies from the employees’ perspectives.

## 3 Materials and methods

### 3.1 Research philosophy

This study adopted an interpretivist paradigm, recognising that quiet-quitting experiences reflect multiple, socially constructed realities rather than objective phenomena (Goldkuhl, 2012). Ontologically, it was assumed that workplace realities vary across contexts and individuals, shaped by personal experiences and organisational cultures. Epistemologically, a constructionist stance was adopted, acknowledging that knowledge emerges through researcher-participant interactions (Kapilima, 2024). The interpretivist paradigm aligned with the research aims by enabling the exploration of diverse perspectives and meanings (Pervin and Mokhtar, 2022). Semi-structured interviews facilitated this interpretive inquiry by allowing participants to articulate their experiences in their own terms whilst enabling the researcher to probe emerging themes.

### 3.2 Research approach

This study employed a qualitative research approach to explore the lived experiences and perceptions of Gen Z and Millennial employees regarding quiet quitting (Creswell and Poth, 2016). Qualitative methodology was selected over quantitative approaches for several reasons. Quiet quitting represents an emerging phenomenon that requires exploratory investigation rather than hypothesis testing. Understanding the nuanced meanings employees ascribe to their disengagement necessitates rich, contextual data that cannot be obtained through surveys alone. In addition, the limited existing literature precluded the development of validated measurement instruments (Taherdoost, 2022). The inductive approach enabled theory development from participants’ experiences rather than testing predetermined hypotheses (Saunders et al., 2019), and was particularly appropriate given the study’s exploratory aims and the nascent state of quiet-quitting research in South African contexts.

### 3.3 Population and sampling

The target population comprised Gen Z and Millennial employees in professional roles in South Africa. From this population, a purposive sample of 12 participants was selected. Non-probability sampling was employed given the need to access participants with specific characteristics and experiences relevant to quiet quitting (Stratton, 2023). Two complementary non-probability sampling techniques were used. Purposive sampling enabled the deliberate selection of information-rich participants who could provide valuable insights on quiet quitting (Thomas, 2022; Turner, 2020). Snowball sampling then facilitated access to additional participants through existing participants’ networks, yielding diverse experiences and perspectives (Creswell, 2023; Dragan and Isaic-Maniu, 2022).

Inclusion criteria required participants to be between 18 and 35 years old and to have a minimum of one year’s working experience in full-time roles. This age range captured Gen Z employees (ages 18 to 27) and younger-to-mid-career Millennial employees (ages 28 to 43), though the sample skewed towards younger Millennials given the 35-year upper limit. The one-year experience requirement ensured that participants had sufficient workplace exposure to engage meaningfully with quiet-quitting concepts. Additional criteria included current employment in South Africa, professional or white-collar roles, and willingness to discuss workplace experiences openly. No restrictions were placed on sector, organisation size, or geographical location within South Africa.

The sample size of 12 participants aligns with established guidance for reflexive thematic analysis in qualitative research. Braun and Clarke (2022) recommend samples of six to 15 participants for studies employing reflexive thematic analysis with a relatively homogeneous population, whilst Guest et al. (2020) demonstrate that thematic saturation in homogeneous samples is typically achieved within 12 interviews. Wutich et al. (2024), in their integrative review of sample sizes across 10 types of qualitative data analysis, further support this range, reporting that thematic saturation in interview-based studies is commonly reached between nine and 17 participants, with smaller samples appropriate when the population shares key characteristics. The present sample’s relative homogeneity, comprising early-career, tertiary-educated South African professionals aged 23 to 31, aligns with these parameters.

Saturation was monitored iteratively throughout data collection through a process of concurrent coding and memoing. Following each interview, the researcher reviewed the transcript and noted emerging codes and patterns. A saturation log was maintained to track the introduction of new codes across successive interviews. By the tenth interview, no substantive new codes or themes were generated, and the final two interviews served to confirm and enrich existing patterns without introducing additional conceptual material. This approach to saturation assessment is consistent with Saunders et al.’s (2018) model of data saturation in qualitative research, which emphasises the cessation of new thematic information as the primary indicator. Participant demographics are detailed in Table 1.

**Table 1:** Participant demographics (*N* = 12).

ID	Age	Gen.	Gender	Race	Exp.	Job title	Sector	Education
P1	28	Mill.	Male	Black	5 years	Corporate commercial adviser	Legal	Honours
P2	25	Gen Z	Male	Black	3 years	Principal engineer	Automotive	Bachelor’s
P3	26	Gen Z	Female	Black	3 years	Quantity surveyor	Construction	Honours
P4	28	Mill.	Female	Black	5 years	Candidate valuer	Construction	Honours
P5	23	Gen Z	Female	Indian	1 year	Quantity surveyor	Construction	Bachelor’s
P6	29	Mill.	Female	Black	2 years	Analyst	Retail	Honours
P7	31	Mill.	Male	Black	7 years	Financial accountant	Retail	Honours
P8	25	Gen Z	Female	Black	1 year	OD intern	Manufacturing	Honours
P9	31	Mill.	Male	Black	5 years	Data analyst	Academia	Master’s
P10	24	Gen Z	Female	Black	2 years	CA trainee	Financial services	Honours
P11	24	Gen Z	Female	Black	1 year	QA officer	Construction	Honours
P12	23	Gen Z	Female	Black	3 years	Property asset manager	Construction	Honours

Mill., Millennial; OD, Organisational Development; CA, Chartered Accountant; QA, Quality Assurance.

The predominance of female participants (75%) and Black participants (92%) reflects both South Africa’s demographic composition and the accessibility of the researcher’s network. The concentration of construction sector participants (42%) emerged through snowball sampling and may limit generalisability but provided rich, sector-specific insights. Most participants held Honours degrees (75%), indicating a relatively educated sample consistent with professional roles. Experience levels ranged from 1 to 7 years (M = 3.17), capturing early-to-mid-career professionals most relevant to quiet-quitting research.

It should be noted that the analysis treats Gen Z and Millennial participants as a combined cohort for two reasons. First, the sample size (seven Gen Z, five Millennial) does not support robust between-group comparison. Second, preliminary analysis indicated substantial overlap in the themes articulated by both groups, with differences manifesting primarily in degree rather than kind. Where the data permitted, generational nuances are noted within the findings. For instance, Gen Z participants (P2, P5, P10) placed relatively greater emphasis on flexibility and remote work as conditions of engagement, whilst Millennial participants (P1, P7, P9) more frequently referenced career progression and financial stability. However, these patterns should be interpreted cautiously given the small sub-group sizes, and future research with larger, balanced samples is needed to test whether these apparent generational differences are robust.

### 3.4 Data collection

Semi-structured interviews were employed as the primary data collection method, enabling exploration of participants’ lived experiences with quiet quitting whilst maintaining consistency across interviews (Alamri, 2019). An interview guide was used to maintain consistency whilst allowing the researcher to probe emerging themes and clarify ambiguities (Adeoye-Olatunde and Olenik, 2021). This balance between structure and flexibility was particularly appropriate for exploring quiet quitting, where individual experiences vary considerably.

Twelve semi-structured interviews were conducted, each lasting between 30 and 55 min (*M* = 42 min). Interviews were conducted remotely via Microsoft Teams (*n* = 7) and Google Meet (*n* = 5) to accommodate participants’ preferences and geographical locations across South Africa. Both platforms’ integrated recording features facilitated automatic audio recording and transcription. The interview guide covered four main areas: (1) participants’ understanding and experiences of quiet quitting, (2) factors contributing to disengagement, (3) observable behaviours and attitudes associated with quiet quitting, and (4) recommended organisational strategies for mitigation. All interview recordings and transcripts were stored securely on password-protected, encrypted devices accessible only to the research team. Transcripts were anonymised immediately upon generation, with participants assigned pseudonyms (P1 to P12) and identifying details removed.

### 3.5 Data analysis

Reflexive thematic analysis was employed to identify, analyse, and interpret patterns within the data (Braun and Clarke, 2021). This approach was selected because it aligns with the study’s interpretivist paradigm by acknowledging researcher subjectivity as integral to meaning-making rather than as bias to be eliminated (Braun and Clarke, 2021), provides systematic procedures ensuring analytical rigour, and allows for interpretive depth (Braun and Clarke, 2022). Analysis followed Braun and Clarke’s (2022) six-phase framework: (1) familiarisation with the data, (2) generating initial codes, (3) searching for themes, (4) reviewing themes, (5) defining and naming themes, and (6) producing the report.

### 3.6 Trustworthiness

To enhance trustworthiness, this study applied the four principles outlined by Lincoln and Guba (1985) and elaborated by Bezuidenhout et al. (2021): confirmability, transferability, dependability, and credibility. Confirmability was supported through systematic coding procedures and the direct quotation of participants’ responses, maintaining transparency between data and interpretation. An audit trail was maintained, documenting analytical decisions including code development, theme refinement, and the rationale for collapsing or separating codes during iterative review. Transferability was enhanced through thick description of the research context, participant characteristics, and methodological procedures, enabling readers to assess the applicability of findings to comparable settings. Dependability was strengthened through a detailed and replicable account of the research design, data collection protocols, and analysis procedures. Credibility was supported through multiple strategies: data triangulation by cross-referencing accounts across participants to identify convergent and divergent perspectives; peer debriefing sessions with research supervisors to challenge interpretive assumptions; and the maintenance of a reflexive journal to document the researcher’s evolving understanding and potential biases throughout the analytical process.

### 3.7 Researcher reflexivity

Consistent with the principles of reflexive thematic analysis (Braun and Clarke, 2021), the researcher’s positionality and its potential influence on the research process are acknowledged. The primary researcher is a young Black South African professional who falls within the Millennial generational cohort and has personal experience of the workplace dynamics under investigation. This positionality afforded several advantages, including ease of rapport with participants, familiarity with the cultural and professional contexts discussed, and sensitivity to the nuances of participants’ language and references. However, it also carried the risk of over-identification with participants’ perspectives and the potential to project personal experiences onto the data. To mitigate these risks, the researcher maintained a reflexive journal throughout data collection and analysis, documenting assumptions, emotional responses, and interpretive decisions. Preliminary codes and themes were discussed with the research supervisors, who provided critical scrutiny from perspectives outside the participant demographic. The researcher also deliberately sought disconfirming evidence within the data, interrogating instances in which participants’ accounts diverged from emerging patterns. These practices ensured that the analysis remained grounded in participants’ accounts rather than the researcher’s assumptions.

### 3.8 Ethical considerations

This study adhered to the requirements of the University of Johannesburg’s Department of Industrial Psychology and People Management Ethics Committee (clearance code IPPM 2024 908[M]). All participants received detailed information sheets explaining the study’s purpose, procedures, potential risks and benefits, data usage, and confidentiality measures before participation. Participants provided written informed consent electronically before interviews, with verbal consent reconfirmed immediately before recording commenced. The consent process emphasised voluntary participation, and participants were free to withdraw at any point without providing reasons. All participants were assigned pseudonyms (P1 to P12) used throughout transcription, analysis, and reporting.

## 4 Findings

This study explored quiet quitting amongst South African Gen Z and Millennial employees, examining contributing factors, behavioural manifestations, and mitigation strategies. Thematic analysis of 12 interviews revealed three interrelated themes: (1) quiet-quitting behaviours and attitudes, (2) workplace value misalignment, and (3) organisational strategies to mitigate quiet quitting. These themes and their constituent sub-themes are summarised in Table 2, with frequency counts indicating how many participants discussed each aspect.

**Table 2:** Summary of findings.

Theme	Description	Sub-themes
Theme 1: Quiet-quitting behaviours and attitudes	Behaviours and attitudes displayed by quiet-quitting employees, limiting participation and contribution in the workplace.	Withdrawal Minimal effort performance Career transition preparation Generational work attitudes Digital distraction
Theme 2: Workplace value misalignment	Disconnect between an employee’s values and those of their organisation leading to quiet quitting.	Inadequate compensation and recognition Limited growth and development opportunities Changing generational priorities Toxic work environment
Theme 3: Organisational strategies to mitigate quiet quitting	Initiatives for overcoming quiet quitting by promoting workplace culture and voice, early detection, well-being and work-life balance, recognition and reward systems, and growth and development opportunities.	Workplace culture and voice Detection and intervention Growth and development opportunities Well-being and work-life balance Recognition and reward systems

### 4.1 Theme 1: quiet-quitting Behaviours and attitudes

This theme revealed that quiet-quitting employees exhibit behaviours and attitudes that limit their participation and contribution in the workplace.

#### 4.1.1 Withdrawal

Participants reflected on how quiet-quitting employees withdrew in the workplace by reducing their contributions to the bare minimum requirements of their jobs. A recurring pattern was increased absenteeism and isolation through non-participation in team activities and events.

*“They’re just not engaged. Like in the WhatsApp group, they would not even know that these WhatsApp groups are for work, or they would not even speak in the WhatsApp groups.”* (P2, 25, 3 years’ experience).

*“Zoning out into their mindset and not being present in the moment, where they are at, in the meetings, with the task that they are doing.”* (P5, 23, 1 year’s experience).

Participants’ accounts suggest that quiet-quitting employees withdraw from the workplace through decreased participation and increased absenteeism. These findings reinforce the view that quiet quitting can be observed through limited interaction in discussions and reduced collaboration (Shah and Parekh, 2023). This observation aligns with Galanis et al. (2023), who asserted that quiet quitting is a practice by which employees psychologically detach in the workplace. The findings also correspond with Srivastava et al. (2025) and Rodríguez-Montoya et al. (2024), who note that quiet quitting can damage workplace relationships, with employees ceasing to communicate with colleagues. Interpreted through job embeddedness theory, withdrawal behaviours indicate a weakening of the links dimension, as employees progressively disengage from the interpersonal connections that would otherwise anchor them to the organisation.

#### 4.1.2 Minimal effort performance

Participants shared accounts of how quiet-quitting employees reduce their work effort to the bare minimum in order to preserve their employment.

*“Just from my experience, it’s when we do things because we need money to cover our needs, so we tend to do the bare minimum in terms of the things we are supposed to do. We tend not to contribute a lot.”* (P1, 28, 5 years’ experience).

*“They try to do the bare minimum and then in the extra time that they have they try to pick up a second job or start a side hustle to make ends meet. Some people are motivated by mental health concerns, they might not do anything else in that extra time, they will just rest.”* (P9, 31, 5 years’ experience).

Participants’ accounts suggest that employees quiet-quit in order to maintain their employment and generate the income needed to meet their basic needs. These findings are supported by Yikilmaz (2023), who stated that quiet-quitting employees do the bare minimum at work to avoid dismissal and for fear of income loss. Notably, this finding stands in tension with the narrative that quiet quitting represents a values-driven rejection of overwork culture. Whilst participants such as P9 described quiet quitting as a deliberate strategy for protecting mental health through boundary-setting, others, including P1, framed it as an economic survival mechanism necessitated by South Africa’s constrained labour market. This duality suggests that quiet quitting is not a unitary phenomenon but rather encompasses at least two distinct motivational orientations: a protective orientation rooted in self-preservation under conditions of economic precarity, and an assertive orientation driven by generational values around well-being and work-life boundaries. This distinction has implications for job embeddedness theory: employees in the protective orientation remain embedded primarily through high perceived sacrifice (the cost of job loss in a high-unemployment context), whilst those in the assertive orientation display low embeddedness across all three dimensions, remaining only because alternatives are not yet secured. This study does not concur with Öztürk et al. (2023), who suggest that quiet-quitting employees do not fear job loss because they perceive it as an opportunity for new career prospects, a position that may reflect labour market conditions substantially different from the South African context, where youth unemployment exceeds 60%.

#### 4.1.3 Career transition preparation

Participants highlighted that a notable indicator of quiet quitting is the desire to leave one’s organisation in search of other opportunities, with quiet-quitting employees demonstrating active job-seeking behaviours.

*“When it comes to things like applying for jobs, they are not even afraid to use the company resources to apply actively. If something better comes, they are not going to even think twice.”* (P4, 28, 5 years’ experience).

*“I think with Gen Z it could be an inflation-related thing, but us wanting to be able to make more money or do more things, whereas older generations are loyal, and so, I guess we job-hop more often because we think the grass is green on the other side, if you feel like you are getting underpaid.”* (P9, 31, 5 years’ experience).

This study found that quiet-quitting employees are often preparing to transition in their careers by demonstrating job-seeking behaviours such as taking calls from recruiters, attending job interviews, and applying for other positions during working hours. These findings confirm those of Chillakuri (2018) and O’Connor and Raile (2015), who stated that Gen Z and Millennial employees are inclined to leave their jobs in pursuit of higher-paying positions. The findings also confirm the prevalence of the post-COVID-19 trend of job-hopping, whereby employees change jobs in a short period to improve their earning potential and work-life balance (Pawar and Pandit, 2023; Saraiva and Nogueiro, 2025). The meaning employees derive from work, particularly in the context of post-pandemic uncertainty, further shapes their attachment to or detachment from formal employment (Jacobs et al., 2024). Within the framework of job embeddedness theory, career transition preparation signals that the sacrifice dimension has been reassessed: employees who actively seek alternatives have concluded that the perceived costs of leaving are outweighed by the anticipated benefits of departure.

#### 4.1.4 Generational work attitudes

Participants provided insight into the ways in which generational differences influence quiet quitting, particularly regarding loyalty and organisational citizenship behaviour.

*“So, for older people, it’s more of settling down and working there until you retire and then you just retire and spend your money. So, for us, we just feel like there’s more opportunities out there. I feel like, I’m comfortable, I need a change, you will start looking for other opportunities out there.”* (P3, 26, 3 years’ experience).

*“The older generations believe that you do not leave your job, you are going to be stuck there. Whereas the new generation, ‘if you do not do things in a way I prefer, I would rather quit.’ The older generation will complain about things, because they believe that you stay in your job, even if it is hard; you stay in it.”* (P7, 31, 7 years’ experience).

The data suggest that employee turnover is higher amongst younger employees than older employees. Participants indicated that younger employees show lower levels of organisational loyalty, and that the higher turnover rate amongst these employees is motivated by the need for improvement and income generation. Lestari et al. (2024) confirm that Gen Z and Millennial employees exhibit low loyalty to their organisations and are likely to change workplaces. These findings connect to the sacrifice dimension of job embeddedness theory, which focuses on the losses that employees would experience if they were to leave their jobs (Mitchell et al., 2001). By contrast, older employees tend to demonstrate greater dedication in the workplace, driven by loyalty to their organisations, consistent with Hoole and Bonnema (2015). Viewed through the theory of generations, this pattern reflects the distinct formative experiences of each cohort: older employees, socialised into workplace cultures that valorised tenure and organisational loyalty, perceive higher sacrifice in departure, whilst younger employees, shaped by economic instability and portfolio career norms, perceive lower sacrifice and higher opportunity costs of remaining.

#### 4.1.5 Digital distraction

Participants highlighted that excessive digital media use by quiet-quitting employees negatively affects efficiency and leads to missed deadlines.

*“So, there’s probably friendships there. So, they talk a lot. They gather and have conversations for hours. And obviously, they always on their phones. Yes, that one. They always on their phones, they do not reach their deadlines.”* (P8, 25, 1 year’s experience).

*“Not being engaged in conversations that are happening at their place of work. If people are communicating about a topic, you choose to take out your EarPods and not engage.”* (P10, 24, 2 years’ experience).

This study found that quiet-quitting employees are more likely to miss deadlines, with participants attributing this partly to excessive social media usage at work. This is consistent with Liu-Lastres et al. (2024), who stated that quiet-quitting employees are likely to increase inefficiencies in the workplace, and with Sarwar et al. (2024), who noted that quiet quitting may manifest as a lack of engagement in tasks and failure to meet deadlines. This contrasts with Celebi and Terkan (2020), who found that high social media usage can improve employee performance by enhancing communication and cooperation.

### 4.2 Theme 2: workplace value misalignment

This theme presents the key reasons for misalignment that contribute to quiet quitting amongst Gen Z and Millennial employees.

#### 4.2.1 Inadequate compensation and recognition

Participants frequently cited inadequate pay relative to workload, and a lack of recognition, as primary motivators for quiet-quitting behaviours.

*“It’s not receiving credit and pay for the work you do. To me, I feel that you push yourself beyond the work you are supposed to be doing, and you do not get thanked, you do not get acknowledged for it. Why would you push yourself? Why would you put yourself in that situation? We do not want to be doing the most for our employers at our own expense.”* (P5, 23, 1 year’s experience).

*“I think it means taking a step back, maybe you do not see any progress in your career and then, as you are looking into it, taking another opportunity and you are reducing your duties or you feel you are not well compensated in the position. Due to that you start taking a step back to try to look for another opportunity.”* (P11, 24, 1 year’s experience).

Within this sample, participants’ accounts suggest that when employees perceive that their compensation does not reflect the work they perform, they are not motivated to go the extra mile and may consequently quiet-quit. This pattern is consistent with the sacrifice dimension of job embeddedness theory (Mitchell et al., 2001): when the perceived rewards of continued engagement are low, the psychological cost of withdrawing discretionary effort is correspondingly reduced. This corresponds with Detert (2023), who states that quiet-quitting employees exert minimal effort when they perceive an imbalance between their contribution and their compensation, and with Serenko (2024), who noted that employees are not motivated to go the extra mile when they feel unfairly compensated. Research by Mabaso and Mathebula (2025) reveals a dual motivation for Gen Z employees, who value external rewards such as compensation and recognition, but whose job satisfaction and loyalty are primarily driven by intrinsic needs, specifically the desire for growth, a sense of purpose, and a wish to make an impact. Interpreted through the theory of generations, this dual motivation reflects the distinctive orientation of cohorts whose formative experiences included both economic precarity and exposure to purpose-driven organisational narratives, creating a simultaneous demand for material security and meaningful work that older cohorts may not have articulated as explicitly.

#### 4.2.2 Limited growth and development opportunities

The absence of clear pathways for growth, or being assigned tasks below one’s capabilities, were identified as significant factors contributing to disengagement.

*“In my experience, even myself, I have quiet-quit because I looked at the work that I was doing; I felt that the work was not what I should be doing and I thought let me just take a step back and look for opportunities out there so that I use the skill set that I have, rather than staying in this position and be stuck for five years without growth.”* (P12, 23, 3 years’ experience).

*“You feel like you are stuck or you hit a plateau in the role that you are in, and the company does not really have a role for you to explore and grow yourself as an individual.”* (P6, 29, 2 years’ experience).

Participants’ accounts indicate that when employees feel stuck in their careers and do not find purpose in their work, they are less likely to contribute to their full potential. Dhanpat et al. (2025) confirm that early-career professionals derive meaning from personal growth. Interpreted through the lens of job embeddedness theory, limited growth opportunities directly diminish the fit dimension by reducing compatibility between the employee’s professional aspirations and the organisation’s capacity to fulfil them, whilst simultaneously lowering the perceived sacrifice of disengagement, as employees recognise fewer developmental assets that would be forfeited by withdrawing effort. This pattern aligns with Onukwuba (2020) and Racolţa-Paina and Irini (2021), who stated that Gen Z and Millennial employees are likely to change jobs in pursuit of career advancement and growth. Viewed through the theory of generations, these findings further suggest that the emphasis on continuous development reflects a generationally distinctive orientation: both Gen Z and Millennial cohorts, shaped by exposure to rapidly evolving digital economies and portfolio career models, expect organisations to provide ongoing learning opportunities as a baseline condition of engagement rather than an exceptional benefit.

#### 4.2.3 Changing generational priorities

The distinct values and priorities of Gen Z and Millennial employees, including work-life balance, mental health considerations, and a preference for flexibility, emerged as significant contributors to quiet quitting.

*“The trend is that money is no longer important. People care about their overall well-being, and that impacts your mental health, your physical health, your work-life balance and your family life.”* (P4, 28, 5 years’ experience).

*“Younger employees want to work from home. They want to have a lot of flexibility and if they are not getting that it might result into quiet quitting. But older generations believe in working from the office.”* (P2, 25, 3 years’ experience).

Within this sample, the data suggest that young employees are no longer primarily motivated by financial reward at work. This is consistent with Maan and Srivastava (2023) and Seqhobane and Kokt (2021), who note that Gen Z employees are self-sufficient and that monetary compensation is not their primary incentive. Participants also indicated that young employees are more receptive to new ways of working, consistent with Mahmoud et al. (2021), whilst older employees tend to prefer maintaining established practices, as noted by Polat et al. (2019), who found that older employees are less inclined to adapt to change and innovation. These generational differences in workplace orientation align with the theory of generations (Mannheim, 1952), suggesting that the formative experiences of each cohort shape fundamentally different expectations regarding the role of work in broader life.

#### 4.2.4 Toxic work environment

Participants agreed that the perpetuation of a toxic work culture by leaders and managers can significantly drive quiet quitting.

*“The more harsh and ruthless leadership lead to quiet quitting. The styles that tend to isolate employees are the ones that encourage quiet quitting. The one that encourages quiet quitting is the autocratic leadership style.”* (P9, 31, 5 years’ experience).

*“It turns out a toxic workplace environment, it could be employees feeling unmotivated or being disrespected by the higher employees and perhaps feeling undervalued and maybe poor management.”* (P6, 29, 2 years’ experience).

Participants’ accounts indicate that a toxic workplace culture can emerge when managers undermine and disrespect their subordinates. This pattern is noteworthy in that Gen Z and Millennial employees value supportive environments that promote respect (Akbar and Gunawijaya, 2022). Authoritative leadership styles, including top-down approaches, dictatorial management, and micromanagement, were found to contribute to quiet quitting. The findings therefore suggest that a shift away from hierarchical leadership styles is necessary for effectively managing Gen Z and Millennial employees (Dhawan, 2025; George, 2024). The data also suggest that favouritism and office politics erode a sense of fairness in the workplace, contributing to quiet quitting, consistent with Al Soqair and Al Gharib (2023) and Uyan and İbin (2025). Within the conceptual framework of this study, toxic environments represent a fundamental breakdown of the fit dimension: when organisational culture is characterised by disrespect and autocratic control, the misalignment with younger employees’ values of authenticity, collaboration, and mutual respect becomes acute, accelerating psychological withdrawal.

### 4.3 Theme 3: Organisational strategies to mitigate quiet quitting

This theme outlines recommendations for steps that organisations may take, tailored to meet the engagement needs of Gen Z and Millennial employees.

#### 4.3.1 Workplace culture and voice

Most participants argued that organisations should create an environment in which employees feel heard and valued by promoting employee voice.

*“They should have an open space for people to voice your opinions in terms of how you feel about the workplace, your mental health, and make it a place where they can communicate the elements within the organisation.”* (P9, 31, 5 years’ experience).

*“I think they need to start with an anonymous survey on employee well-being. It should be centred around how employees feel about engagement.”* (P8, 25, 1 year’s experience).

Participants suggested that providing employees with opportunities to communicate their concerns plays a significant role in reducing quiet quitting. Recommendations included establishing public forums, implementing anonymous feedback platforms, and adopting open-door policies. These findings correspond with Jelača and Golubović (2024), who suggested that quiet quitting may be mitigated by considering employee feedback, and with Campton et al. (2023), who advocated for open and transparent communication as a means of engaging and retaining employees.

#### 4.3.2 Growth and development opportunities

Participants emphasised the importance of clear career advancement pathways, mentorship programmes, and learning experiences.

*“What fulfils the employees is having a sense of being seen for their efforts of being recognised for into the company, as well as having proper guidance in terms of mentorship and leadership, having a proper manager or someone who the young employee reports to.”* (P2, 25, 3 years’ experience).

*“I would say us Gen Zs are likely to be in a role not based on remuneration but based on the actual environment, the culture, the team, as well as growth, where we are able to learn from those people.”* (P10, 24, 2 years’ experience).

Participants noted that mentors play a crucial role in helping young employees navigate the workplace by providing advice and guidance, consistent with Peretomode and Ikoya (2019), who identified mentorship as a critical mechanism for facilitating skills and knowledge transfer. These findings also align with Racolţa-Paina and Irini (2021), who note that Gen Z and Millennial employees value clear career paths that allow them to develop their skills.

#### 4.3.3 Recognition and reward systems

Participants agreed that a combination of rewards and recognition is important for effectively motivating employees and mitigating quiet quitting.

*“I think the messaging is: ‘I want my money; give me my money’; pro money, so therefore if you are in that standard of older people that you need to work and take your time and build yourself up to start earning a lot of money. With young people we want money from the get-go.”* (P9, 31, 5 years’ experience).

*“Sometimes in some jobs they do that thing of selecting a best employee of the month. I feel like it also encourages people to work hard because you will want to get that certificate cause it’s going to be good on your* CV.*”* (P3, 26, 3 years’ experience).

Participants’ accounts suggest that both monetary incentives and acknowledgement motivate employees. Equitable and competitive salaries can help reduce the incidence of quiet quitting, whilst recognising employees enhances engagement by making them feel valued for their contributions. These findings are consistent with Krishna and Agrawal (2024), who stated that younger employees are motivated by financial rewards and place a high value on recognition.

#### 4.3.4 Well-being and work-life balance

Participants identified the prioritisation of employee well-being as crucial for mitigating quiet quitting.

*“Counselling and therapy, for sure. Mental health is important here for sure. Having physical exercises is always good for mental health. It helps with the office cohesion and how people get along.”* (P1, 28, 5 years’ experience).

*“I think having flexibility in work hours as well, I think, is a big thing. Being able to maybe go home to ditch traffic or having that flexibility and working from home is a big thing.”* (P3, 26, 3 years’ experience).

Wellness initiatives aimed at supporting collective employee fitness were found to strengthen relationships amongst employees and foster cohesion. This aligns with Duda and Drożdżyńska (2024), who suggested that wellness initiatives could mitigate quiet quitting and assist with managing workplace mental health concerns (Dhanpat et al., 2025). Participants’ strong preference for flexible working arrangements aligns with Kgarimetsa and Naidoo (2024) and Rozlan and Subramaniam (2020), who indicated that younger employees are encouraged by such flexibility.

#### 4.3.5 Detection and intervention

Participants highlighted the importance of proactive monitoring and early intervention strategies.

*“Having one-on-one sessions. Why is someone not participating in social events? Why is someone not participating in meetings? Is someone leaving early?”* (P7, 31, 7 years’ experience).

*“This might be a tough one from reviewing work; the work that I do is reviewed by someone. If someone sees that I am not putting a lot of effort into my work, I am just doing the bare minimum, that might be a trigger or an indicator that I am quiet-quitting.”* (P1, 28, 5 years’ experience).

The data highlight the importance of individual consultations with employees as an early intervention for quiet quitting, consistent with Kachhap and Singh (2024), who highlighted the value of management-employee dialogues for understanding employee expectations and needs. Within this sample, the data indicate that a sudden decline in the quality and quantity of work can signal quiet quitting, connecting to Yikilmaz (2023). An opposing perspective offered by Priya et al. (2025) suggests that quiet quitting does not necessarily influence employee performance levels.

## 5 Discussion

This study set out to explore the factors contributing to quiet quitting amongst South African Gen Z and Millennial employees, alongside the organisational strategies needed to address the phenomenon. The findings provide empirical grounding for three interrelated dimensions of quiet quitting: how it manifests behaviourally, what drives it, and how it can be mitigated.

The behavioural manifestations identified in Theme 1 extend the existing literature by adding career transition preparation and digital distraction as observable indicators not previously foregrounded in the South African context. The withdrawal and minimal effort performance sub-themes replicate findings from international studies (Galanis et al., 2023; Yikilmaz, 2023), confirming that these patterns are consistent across national contexts. The prevalence of job-seeking behaviours during working hours aligns with broader post-COVID-19 trends of job-hopping and suggests that quiet quitting and career mobility are interconnected rather than mutually exclusive.

Theme 2 highlights that the root cause of quiet quitting is not simply dissatisfaction with a single factor, but rather a broader misalignment between the values and expectations of younger employees and those of their organisations. The interplay between inadequate compensation, limited growth, generational priorities, and toxic environments maps directly onto the fit and sacrifice dimensions of job embeddedness theory (Mitchell et al., 2001). Specifically, the findings suggest that when employees perceive low organisational fit, characterised by misalignment between personal values and organisational culture, the psychological threshold for disengagement is substantially reduced. Importantly, the data extend Mitchell et al.’s (2001) original formulation by revealing that for Gen Z and Millennial employees, the links dimension operates differently than for older cohorts: participants described workplace connections as contingent and instrumental rather than as anchoring ties that constrain departure. This suggests that the protective function of organisational links against turnover-related behaviours may be attenuated amongst younger workers whose social capital is distributed across digital and professional networks beyond a single employer. These findings contribute a generational refinement to job embeddedness theory, proposing that the relative weight of the three dimensions shifts across cohorts, with fit assuming primacy and links diminishing in salience for younger employees.

The findings also add nuance to the generational differences literature. Participants consistently distinguished their own priorities, including flexibility, recognition, mental health, and career advancement, from those they attributed to older generations. This is consistent with the theory of generations (Mannheim, 1952) and its application to workplace behaviour, indicating that Gen Z and Millennial employees respond to disengagement conditions in ways that reflect their cohort’s formative experiences and values.

Theme 3 demonstrates that employees themselves have clear ideas about what organisational interventions would address their disengagement. Dhanpat et al. (2018) suggest that deploying self-management strategies can assist in this regard. The prominence of workplace culture and voice as the most frequently cited sub-theme (*n* = 14) highlights that being heard is perceived by younger employees as a fundamental organisational responsibility rather than merely a beneficial add-on. This finding supports calls for a shift in HRM towards employee experience design and strategic HR analytics (Falletta and Combs, 2021; Jacobs et al., 2024).

The proposed quiet-quitting framework developed from the findings presents four operational phases: identifying contributing factors, behavioural and attitudinal observation, action, and maintenance. This framework provides a structured approach for HR practitioners and leaders to move from detection to sustained intervention. It positions quiet quitting not as a problem to be solved once, but as an ongoing management responsibility requiring continuous monitoring and adaptation.

### 5.1 Theoretical contributions

This study makes three contributions to the theoretical understanding of quiet quitting. First, it offers a generational refinement of job embeddedness theory (Mitchell et al., 2001) by demonstrating that the relative salience of the three embeddedness dimensions, namely fit, links, and sacrifice, varies across generational cohorts. Participants’ accounts consistently indicated that organisational fit, understood as value alignment and developmental compatibility, was the dominant determinant of engagement, whilst organisational links played a diminished role relative to the patterns described in studies of older workforces. This suggests that for Gen Z and Millennial employees, whose professional identities and social networks extend beyond organisational boundaries, the links dimension may function as a weaker retention mechanism than previously theorised.

Second, the findings introduce a motivational typology of quiet quitting that distinguishes between protective and assertive orientations. The protective orientation, characterised by effort reduction as an economic survival strategy under conditions of labour market constraint, differs qualitatively from the assertive orientation, in which employees deliberately set boundaries to protect well-being and assert generational values. This distinction advances understanding beyond unitary conceptions of quiet quitting and carries implications for how organisations diagnose and respond to disengagement.

Third, the study extends the theory of generations (Mannheim, 1952) by providing empirical evidence from a non-Western, emerging economy context. Whilst generational theory has been critiqued for its Western-centric foundations, the present findings suggest that generational patterns in workplace orientation are discernible amongst South African professionals, albeit shaped by context-specific factors such as extreme youth unemployment, post-apartheid workplace dynamics, and the economic precarity that distinguishes the South African labour market from the developed-economy settings in which most generational research has been conducted.

## 6 Implications for practice

### 6.1 Implications for HR professionals

HR professionals need to understand the employee experience within their organisations in order to take proactive measures to create an engaging workplace. The study identified key early signs of quiet quitting, including a subtle decline in the quality and output of work, alongside other indicators such as withdrawal, minimal effort performance, career transition preparation, differences in generational work attitudes, and digital distraction. By understanding these indicators, HR professionals can contribute significantly to the early detection of quiet behaviours and attitudes, helping to mitigate quiet quitting and its negative consequences. The data also suggest the potential value of retention and organisational strategies tailored to engage Gen Z and Millennial employees, including growth and development opportunities, remote work options, well-being and work-life balance, and recognition and reward systems.

### 6.2 Implications for leadership

Leaders may benefit from exercising leadership styles and practices that enhance employee morale, motivation, and engagement. Creating a safe environment, active listening, and employee feedback through workplace culture and voice are crucial. Leaders require training in adaptive management skills that resonate with younger employees and enable them to identify signs of quiet quitting. Participants expressed a strong desire to be heard by their leaders. Empowering employees is likely to foster engagement and a stronger sense of being valued.

### 6.3 Organisational implications

Organisations may benefit from preparing and adapting their ways of working and values to meet the distinct needs and expectations of Gen Z and Millennial employees effectively. These adaptations include open-door policies, employee forums, and regular feedback sessions. Organisations could consider prioritising equipping leaders and HR professionals with the skills needed to mitigate quiet quitting in the workplace by evaluating and reforming their leadership development programmes.

## 7 Limitations

Despite the valuable insights generated, several limitations are acknowledged and warrant careful consideration. The actual age range of participants (23 to 31 years) did not span the full inclusion criterion of 18 to 35 years, meaning that the experiences of the youngest Gen Z employees (18 to 22) and older Millennials (32 to 35) remain unrepresented. The results should therefore be interpreted with caution and cannot be generalised to other generational cohorts or to the full age spectrum within the target cohorts.

The predominance of female participants (75%) and Black participants (92%), whilst partially reflective of South Africa’s demographic composition, was primarily shaped by the accessibility of the researcher’s professional and academic networks. This concentration may have foregrounded experiences and workplace dynamics more commonly encountered by Black women in professional roles, potentially underrepresenting the quiet-quitting experiences of male employees, employees from other racial groups, and those in less urbanised settings. Similarly, the concentration of construction sector participants (42%), which emerged through snowball sampling, may have introduced sector-specific dynamics, such as hierarchical project structures and site-based supervision, that do not generalise to other industries.

The sample’s educational profile, with 75% holding Honours degrees, further limits transferability, as the findings primarily reflect the experiences of urbanised, tertiary-educated, young professionals in white-collar roles and may not capture the disengagement patterns of employees in blue-collar, informal, or less credentialled employment contexts. The qualitative design, whilst appropriate for exploratory inquiry, does not permit causal claims or statistical generalisation. These limitations are acknowledged transparently, and caution is advised in extending the findings beyond the population from which the sample was drawn.

## 8 Recommendations for future research

There is limited research on the factors that drive quiet-quitting behaviours amongst Gen Z and Millennials in the South African context (Bangura and Lourens, 2023; Utete et al., 2023), and greater depth of academic inquiry is required. Future research should employ longitudinal designs to track changes in quiet-quitting behaviours over time and assess the effectiveness of mitigation strategies. Quantitative and mixed-methods studies are needed to validate the themes identified in this qualitative research and to establish the prevalence of quiet quitting across different contexts.

Broadening the scope of this research through comparative cross-industry analysis, including a wider variety of Gen Z and Millennial employees from fields such as technology and healthcare, both of which are susceptible to quiet quitting (Duda and Drożdżyńska, 2024; Kang et al., 2023), would be advantageous. Such an approach would enable future research to explore how the factors contributing to quiet quitting vary or remain consistent across different industries.

## 9 Conclusion

This study addressed gaps in quiet-quitting research by exploring the phenomenon amongst South African Gen Z and Millennial employees. Through qualitative inquiry that privileged participants’ voices, the study identified behavioural manifestations, contributing factors, and mitigation strategies specific to younger workers in an emerging economy context. The findings suggest that organisational factors, particularly the perceived mismatch between the values and expectations of younger employees and those of their employing organisations, play a notable role in quiet quitting within this sample.

Based on these findings, a quiet-quitting framework was proposed that combines contributing factors with actionable solutions. Offering flexible work arrangements, cultivating an inclusive workplace culture, recognising employees, ensuring fair compensation, and maintaining open-door leadership practices are amongst the proposed actions. These strategies may assist organisations in taking a proactive approach to detecting and managing quiet quitting, thereby potentially enhancing employee engagement and organisational performance. In doing so, this study contributes to the growing body of evidence on quiet quitting and offers a contextually grounded perspective that complements the predominantly Western literature on this emerging workplace phenomenon.

## Data Availability

The original contributions presented in the study are included in the article/supplementary material, further inquiries can be directed to the corresponding author.
